# Sociodemographic and Perinatal Correlates of Perceived Obstetric Violence in Spain: A Cross-Sectional Study Informed by an Intersectional Framework

**DOI:** 10.3390/nursrep16100366

**Published:** 2026-10-15

**Authors:** Desirée Mena-Tudela, Josefina Goberna-Tricas, Susana Iglesias-Casás, Irene Llagostera-Reverter, Ainoa Biurrun-Garrido

**Affiliations:** 1 Nursing Research Group (Grupo de Investigación Enfermería, GIENF Code 241), Department of Nursing, Faculty of Health Sciences, Instituto Universitario de Estudios Feministas y de Género Purificación Escribano, Universitat Jaume I, Avda. Sos I Baynat s/n, 12071 Castellón, Spain; 2 Department of Public Health, Mental Health and Maternal and Child Health Nursing, ADHUC—Research Center for Theory, Gender, Sexuality, University of Barcelona, Gran Via de les Corts Catalanes, 585, Eixample, 08007 Barcelona, Spain; jgoberna@ub.edu; 3 Centro de Salud Adeje, Gerencia de Atención Primaria, Servicio Canario de Salud, 38670 Santa Cruz de Tenerife, Spain; matronasu@gmail.com; 4 Nursing Research Group (Grupo de Investigación Enfermería, GIENF Code 241), Department of Nursing, Faculty of Health Sciences, Universitat Jaume I, Avda. Sos I Baynat s/n, 12071 Castellón, Spain; 5 Research Group Dolor, Actividad Física, Nutrición y Salud (DAFNis), Departament d’Infermeria de Salut Pública, Salut Mental i Maternoinfantil, Facultat D’infermeria, Universitat de Barcelona, 08036 Barcelona, Spain; ainoabiurrun@gmail.com

**Keywords:** obstetric violence, intersectional framework, sociodemographic factors, abuse, childbirth, disrespect, nursing

## Abstract

**Background/Objectives**: The aim of this study is to analyse the association between sociodemographic and obstetric characteristics and perceived or suspected obstetric violence in Spain, informed by an intersectional framework. **Methods**: A cross-sectional study was conducted using a non-probabilistic online survey of women who gave birth in Spain between March 2020 and April 2021. “Yes” and “do not know” responses were combined as perceived or suspected obstetric violence. Descriptive and bivariate analyses (chi-squared and Fisher’s exact tests) were followed by a multivariable binary logistic regression including, a priori, age, occupation, educational level, social class, ethnicity, type of birth, type of healthcare and autonomous community. **Results**: Of 6060 women, 33.5% (*n* = 2030) reported or suspected obstetric violence. In the adjusted model (*n* = 5978; *Nagelkerke R*^2^ = 0.071), the outcome was associated with all variables except social class. Odds were higher after urgent caesarean section (*aOR* = 2.16) and instrumental birth (*aOR* = 2.76) than after vaginal birth, and lower in private than in public healthcare (*aOR* = 0.67). Estimates for ethnic minorities were very imprecise because of the small size of these groups. **Conclusions**: Perceived or suspected obstetric violence was associated with several sociodemographic, obstetric and healthcare characteristics, most strongly with type of birth, although the explanatory capacity of the model was low. These findings support reviewing interventionist obstetric practices and using sampling strategies that include minority groups.

## 1. Introduction

Obstetric violence (OV) is emerging as a phenomenon of great importance in the area of perinatal care [1], from its origin as a term in the 2007 “Organic Law on the Right of Women to a Life Free of Violence” in Venezuela [2]. This concept covers the appropriation of women’s bodies and reproductive processes by health personnel, characterized by dehumanizing treatment, abuse of medication and pathologization of physiological processes. This results in loss of women’s autonomy and decision-making capacity regarding their bodies and sexuality, thus negatively affecting their quality of life [2,3] and violating their sexual and reproductive rights [4]. OV appears in various forms, from physical and psychological violence to verbal abuse and humiliation during visits [5]. The denial of care, lack of privacy and application of procedures without consent are concrete examples of this violence, with consequences that range from effects on mental health to others at the physical level or the bond with the new-born child, affecting maternal morbidity, mortality, and neonatal and infant development [6]. Of late, some publications have mentioned the progression of the term “obstetric violence” in academic and legislative contexts, highlighting its expansion to cover a wider range of obstetric procedures [7,8]. They point out that although the term may have emotional connotations and suggest intentionality on the part of health care providers, the confusion often arises from systemic problems or lack of training. Consequently, some authors propose use of “obstetric abuse” as a more comprehensive term that recognizes diverse forms of mistreatment and negligence [8,9], although the imprecision of this term means that it does not fully cover the institutionalized, structural, cultural problem that OV represents [10,11,12].

The urgent need to address OV has gained international recognition, as evidenced in the United Nations report on violence against women. This recognition emphasizes the importance of combating OV as a violation of the fundamental right of women to receive respectful care and threatening their right to life, health and bodily integrity [13]. Understanding the sociodemographic dynamics that influence the perception and experience of this violence is crucial to addressing its causes and consequences. It is essential to understand OV within the wider framework of oppression and discrimination, as illustrated by the case of Argentina [14]. In this sense, the adoption of an intersectional approach is necessary to dismantle systemic structures of violence and oppression as a valuable tool in both feminism and other non-feminist theories [15].

Intersectionality, a theoretical framework proposed by Kimberlé Crenshaw [16], recognizes the interconnections between different forms of oppression related to, among others, gender, race, social class, sexual orientation, gender identity and physical capability. This approach appreciates that people can face multiple forms of oppression simultaneously, the experience of which is unique and complex. By considering obstetric violence from an intersectional perspective, awareness that women experience specific forms of obstetric violence based on their intersectional identities can be achieved. The intersectional perspective allows us to comprehend how obstetric violence differentially affects women and how these experiences are shaped by the intersections of gender, race, social class, sexual orientation, disability, and other factors.

Internationally, studies have revealed the prevalence of OV in different contexts, drawing attention to the pressing need to address this issue. Alarming data, including more than a third of women reporting OV during childbirth in a Spanish context [17,18], underscore the magnitude of the challenge. OV is not limited to particular regions, with significant prevalences observed in European countries, Latin America and the United States [14,19,20,21].

Despite growing global awareness of OV and requests to carry out research in Spain by organizations such as the United Nations [22,23,24], there is a scarcity of comprehensive studies examining this phenomenon in the Spanish context from an official institutional perspective. Thus, existing studies come from different research groups. International literature indicates that age, race, and low socioeconomic status are factors associated with perceiving more obstetric violence [25]. This may be replicated in the Spanish context; however, comparative literature on this is lacking. Based on this literature, we expected that younger age, belonging to an ethnic minority and a less favourable socioeconomic position would each be associated with a higher perception of obstetric violence, independently of the other characteristics examined. This gap in the literature highlights the importance of investigating and comprehending the relationship between the axes of intersectionality, and the perception of obstetric violence in Spain. The study described below attempts to fill this gap and contribute to more holistic understanding of OV at the national level. This study aimed to analyse the association between sociodemographic and obstetric characteristics and perceived or suspected obstetric violence in Spain, informed by an intersectional framework.

## 2. Materials and Methods

### 2.1. Design and Participants

A cross-sectional study was conducted using an online questionnaire. Sample selection was carried out in a non-random manner, through non-probabilistic sampling. Participants could access the online survey if they had given birth in Spain between March, 2020 and April, 2021. The questionnaire remained open between March and April 2021. The study period therefore coincided with the COVID-19 pandemic. Questionnaires that did not enter the birth date correctly were excluded from the analysis. To avoid duplicate responses, the survey was configured to accept only one response per respondent (the “limit to 1 response” option of Google Forms, which requires signing in with an e-mail account), and respondents had to accept the informed consent before accessing the questionnaire. Each response therefore referred to a single birth, and each woman contributed one observation to the analysis. Women who had given birth twice within the eligibility window (13 months) could report only one of the births, so the second one was not collected; this situation was expected to be very rare.

For the purpose of the study, an online questionnaire was hosted on the Google Forms platform. A previously designed instrument for the measurement of obstetric violence in Spain was used as the basis for the questionnaire [17,26,27]. To this, the research team added sociodemographic variables (age, occupation, education, social class, ethnicity, and Autonomous Community), along with obstetric variables such as (type of birth) to examine their associations with perceived or suspected obstetric violence, with the selection and interpretation of sociodemographic variables informed by an intersectional framework. The data analysed in the present study come from the same survey as a previous publication by our group that described obstetric violence in Spain during the COVID-19 pandemic [18]; the present article is therefore a secondary analysis of that dataset. There is no overlap in participants with our earlier studies [17,26,27], which provided the instrument on which the questionnaire was based. The previous publication [18] was a descriptive study that aimed to describe women’s perceptions of obstetric violence, medicalization and interventionism at childbirth in Spain during the SARS-CoV-2 pandemic. The present study addresses a distinct research question: it examines mutually adjusted sociodemographic, obstetric, healthcare and regional correlates of perceived or suspected obstetric violence in a multivariable model informed by an intersectional framework. These adjusted associations and the intersectionality-informed analytical approach were not reported in [18].

The questionnaire was subjected to an initial pilot test with 10 women who were recruited in February 2021 to check the clarity and comprehension of the variables presented. Minimal semantic modifications to the items were required by the participants. The pilot study showed that the total time required to complete the questionnaire was approximately 10 min. The pilot responses were discarded and are not included in the study sample.

Women were recruited through social networks. Some prominent birth support groups in Spain assisted with dissemination, including the Obstetric Violence Observatory and the Birth is Ours association.

### 2.2. Variables

Sociodemographic variables included age, categorized as 18–25, 26–35, 36–45, and >45 years; occupation, categorized as housewife, student, unemployed, employed, self-employed, or other; educational level, categorized as primary, secondary, or third-level education; social class, categorized as low, middle, or high; and ethnicity, categorized as White/Caucasian, Romani, Black, or other. Healthcare and obstetric variables included type of healthcare (private, public, or both), type of birth (scheduled caesarean section, urgent caesarean section, instrumental birth, or vaginal birth), and the Autonomous Community in which obstetric care was provided. Perceived obstetric violence was recorded using three response categories: no, I do not know/no answer (NK/NA), and yes. NK/NA was a single response option of the questionnaire (in Spanish: “No lo sé/No contesta”) that respondents selected actively; it does not correspond to missing data. The exact item was: “Would you say that you have suffered obstetric violence during your obstetric care?” (in Spanish: ¿Dirías que en tu proceso has sufrido violencia obstétrica?). Conceptually, obstetric violence represents a structural and highly normalized form of gender-based violence [25,28]. Because routine or medicalized mistreatment during childbirth is frequently normalized, some women may not identify their experiences as obstetric violence, and an NK/NA response may reflect, at least in part, this lack of recognition [17,29]. It may also reflect reluctance to answer a sensitive question, limited information about what constitutes obstetric violence, or genuine ambivalence. As this option does not allow these reasons to be distinguished, we cannot assume that all women who selected it suspected obstetric violence. Therefore, for the analyses, the variable was dichotomized into “yes or suspected” (yes + NK/NA) and “no”, in order to avoid classifying women who did not explicitly rule out obstetric violence as not having experienced it. “Perceived or suspected obstetric violence” is thus used as an operational label for this combined category, which should be interpreted as obstetric violence reported or not ruled out by the respondent, not as confirmed exposure or as a direct measure of suspicion. Of the 6060 participants, 26.0% (n = 1578) answered ‘yes’ and 7.5% (n = 452) answered ‘do not know’. For the primary analysis, these categories were combined, resulting in 33.5% (n = 2030) being classified as reporting or expressing uncertainty about obstetric violence. To assess the influence of this decision, sensitivity analyses were performed (see Section 2.3 and Supplementary Table S1). The distribution of the three original response categories is shown in Supplementary Table S2.

### 2.3. Statistical Analysis

Firstly, a descriptive analysis of all the variables was carried out, with frequency and percentage. Secondly, a bivariate analysis was performed using the Chi-Squared test through contingency tables between the sociodemographic and clinical variables and perceived or suspected obstetric violence (dichotomized as described above). For ethnicity, because some cells had expected frequencies below 5, statistical significance was assessed using Fisher’s exact test. No correction for multiple comparisons was applied. Differences in the proportion of women reporting or suspecting obstetric violence across Autonomous Communities were assessed using Pearson’s chi-square test of independence. The percentage of women reporting or suspecting obstetric violence in each autonomous community was presented with 95% confidence intervals (normal approximation).

An intersectional framework informed the a priori selection and interpretation of the sociodemographic variables examined. The sociodemographic variables available in the dataset that were conceptually relevant to this framework (age, occupation, educational level, social class, ethnicity and autonomous community), together with key clinical characteristics (type of birth and type of healthcare), were included in the analysis, irrespective of their statistical significance in bivariate analyses or their contribution to model performance. However, given the very small sample sizes within several minority categories and the resulting sparsity of intersectional strata, the feasibility of estimating intersectional effects was assessed. This assessment identified 157 intersectional strata, with a median of two participants per stratum. Of these, 100 strata contained fewer than five participants, including 53 strata with only one participant. Together with the limitations identified in exploratory interaction models, these findings indicated that the available data were insufficient to support reliable estimation of intersectional effects. Consequently, the multivariable analysis was not intended to statistically estimate intersectional effects, and interaction terms or combined intersectional strata were not included in the final model. Accordingly, the intersectional framework informed the selection and interpretation of sociodemographic variables, but the statistical analysis was limited to estimating mutually adjusted associations rather than intersectional effects. Instead, a multivariable binary logistic regression model was fitted for explanatory rather than predictive purposes, entering all these predictors simultaneously, to estimate mutually adjusted associations between the selected sociodemographic and clinical characteristics and perceived or suspected obstetric violence. The dependent variable was coded as absence of perceived obstetric violence (0) versus perceived or suspected obstetric violence (1), the latter including “yes” and “do not know” responses; the event of interest was therefore perceived or suspected obstetric violence.

Categorical variables were entered using dummy coding, with the following reference categories: 26–35 years for age, employed for occupation, third-level education for educational level, middle class for social class, White/Caucasian for ethnicity, vaginal birth for type of birth, public healthcare for type of healthcare, and Catalonia for Autonomous Community. The model was estimated using complete-case analysis and included 5978 participants. A total of 82 participants were excluded due to missing data on age (n = 31) or ethnicity (n = 51), with no overlap between these groups. Results were expressed as adjusted odds ratios (aORs), with their 95% confidence intervals (95% CIs) and *p*-values. An aOR greater than 1 indicated higher odds of perceived or suspected obstetric violence compared with the reference category.

Overall model significance was assessed using the omnibus test, and its explanatory capacity was evaluated using Nagelkerke’s R^2^. Model fit was assessed using the Hosmer–Lemeshow test. Multicollinearity was evaluated using the generalized variance inflation factor (GVIF), with the adjusted GVIF [GVIF^(1/(2 × df))] used to allow comparison across categorical predictors with different degrees of freedom. Because the model was explanatory, classification performance measures such as sensitivity, specificity, and the area under the ROC curve were not used for its evaluation. As a sensitivity analysis, the multivariable binary logistic regression was refitted after excluding participants who selected “do not know/no answer” (NK/NA) for the obstetric violence item. In this analysis, the outcome was coded as 0 = “no” and 1 = “yes”. The same predictors, reference categories, and modelling strategy as in the primary analysis were retained. This analysis was performed to assess whether the main findings were robust to the inclusion of NK/NA responses in the primary outcome. Data were processed using Jamovi 2.3.28 and graphs created using SPSS version 27. Statistical significance was set at *p* < 0.05, two-sided.

### 2.4. Ethical Considerations

Ethics approval and consent to participate: This study was designed in accordance with the principles of the Declaration of Helsinki and under the Organic Law 03/2018 on Personal Data Protection and Guarantee of Digital Rights. In addition, this study received ethical approval from ethics committee of the Univesitat Jaume I (CD/06/2021); Date: 18 February 2021. The data were obtained through a national online survey. Informed consent was obtained from all subjects. The first question of the survey asked women to confirm consent for their participation in the study.

## 3. Results

A total of 6270 responses were obtained (pilot responses excluded), of which 3.35% (*n* = 210) were excluded for not responding to the date of birth within the research period.

### 3.1. Descriptive Analysis: Description of the Study Sample

A total of 6060 participants were included in the study with the most prevalent age range between 26 and 35 years (58.1%; *n* = 3502), the majority of the sample consisting of women in employment (73%; *n* = 4423), with third level education (78.2%; *n* = 4736), middle class (93.5%; *n* = 5664), white or Caucasian ethnicity (98%; *n* = 5887), treated in public health (58.9%; *n* = 3572), and with vaginal delivery (64.1%; *n* = 3882). Some 33.5% (*n* = 2030) reported or suspected having experienced obstetric violence. The Autonomous Community that generated the greatest number of responses was Madrid (15.7%; *n* = 949). Table 1 shows a representation of percentages by study variable.

### 3.2. Bivariate Analysis: Obstetric Violence Related to Obstetric and Sociodemographic Variables

Of the total number of women who responded to the survey, it was observed that those aged between 18 and 25 years reported perceiving more OV, affirming it in 43.1% (*n* = 50) of cases. Regarding occupation, students reported more OV (49.1%; *n* = 27), while women of Romani ethnicity reported the most OV (75%; *n* = 6). Those treated in private healthcare perceived less OV than those in public healthcare, with 27.7% (*n* = 162) of women. With respect to type of delivery, an important difference in the perception of OV was observed between those women whose labour ended in urgent C-section (44.2%; *n* = 404) compared with those who delivery ended with a scheduled C-section (32.4%; *n* = 128). Despite this, those whose labour ended with an instrumental delivery perceived the most OV (49.9%; *n* = 434). Table 2 shows the bivariate analysis where it can be observed that the variables mentioned present significant differences (*p* < 0.05), while education and social class revealed no statistically significant differences.

**Figure 1:**
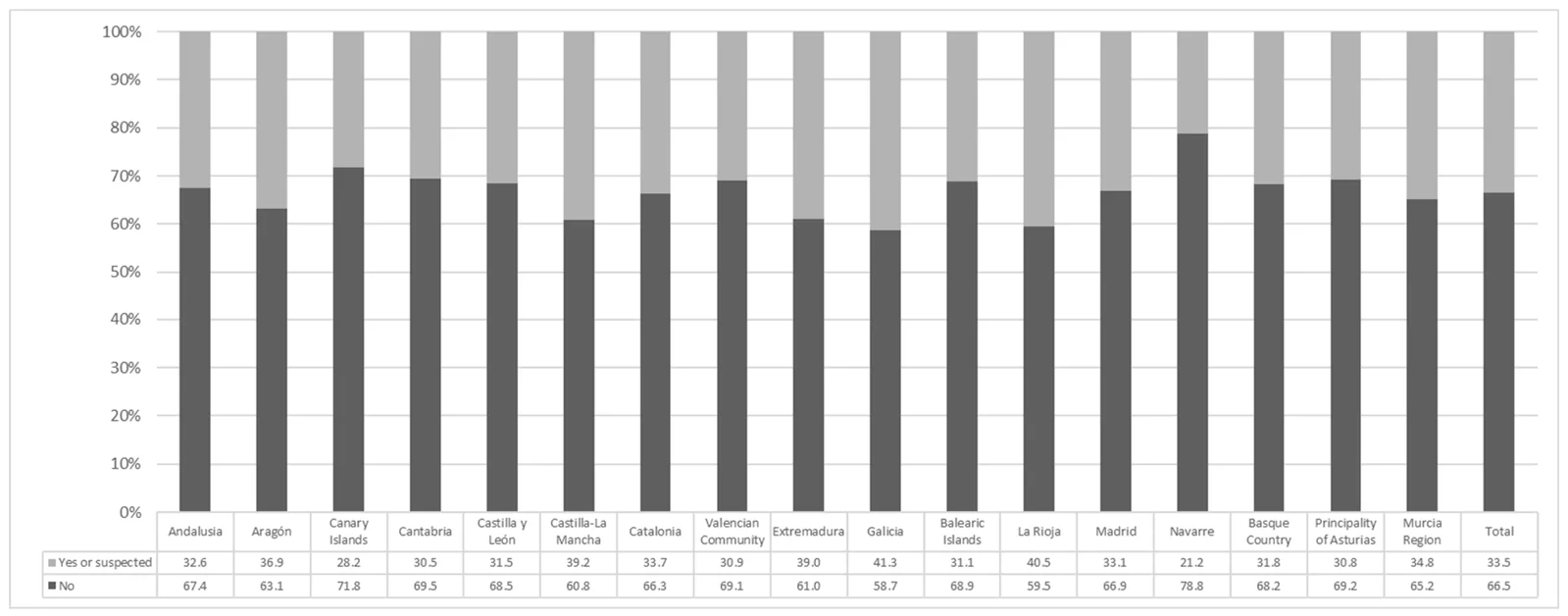
Distribution of OV frequencies by Autonomous Community.

**Figure 2:**
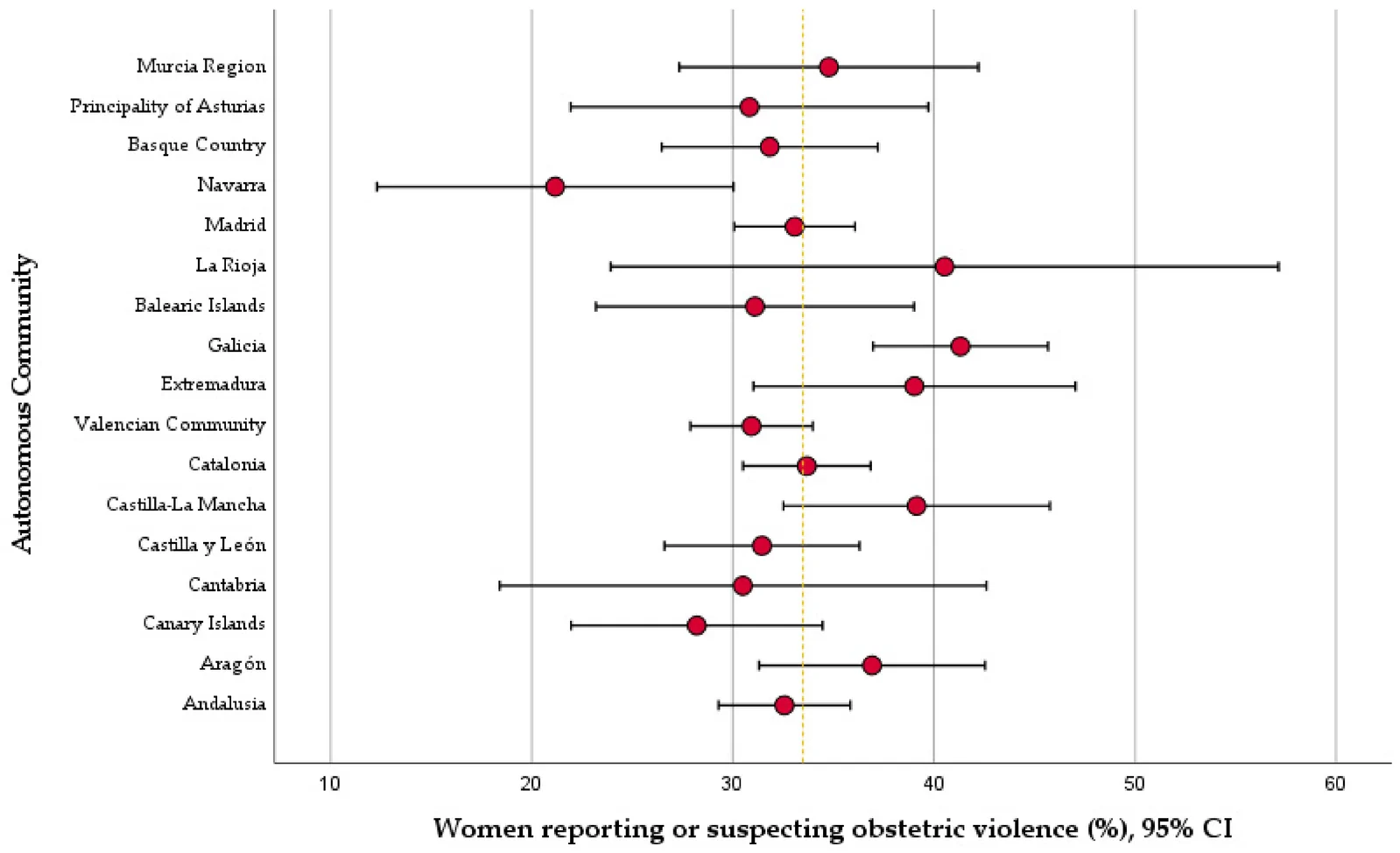
Percentage of women who reported or suspected obstetric violence, by autonomous community, with 95% confidence intervals. Dots represent the percentage in each community; horizontal lines show 95% confidence intervals (normal approximation). The dashed vertical line indicates the overall percentage for Spain in the sample (33.5%).

Concerning how women perceive having experienced OV according to geographical area, some regions can be seen to have a higher perception of OV, led by Galicia (41.3%; *n* = 205) and La Rioja (40.5%; *n* = 15), showing statistically significant differences (*p* = 0.005) (Figure 1 and Figure 2).

### 3.3. Multivariable Results

The multivariable binary logistic regression model was estimated using 5978 participants with complete data and simultaneously included age, occupation, educational level, social class, ethnicity, type of birth, type of healthcare, and Autonomous Community. The overall model was statistically significant (*χ*^2^ (36) = 311.762; *p* < 0.001) and yielded a Nagelkerke R^2^ of 0.071. The Hosmer–Lemeshow test showed no evidence of lack of fit (*χ*^2^ (8) = 10.297; *p* = 0.245). Adjusted GVIF values were close to 1 for all predictors, indicating no relevant multicollinearity. After adjustment for the remaining variables, statistically significant overall associations with perceived or suspected obstetric violence were observed for all predictors except social class (*p* = 0.265).

Among sociodemographic characteristics, significant differences were observed according to age, occupation, educational level, and ethnicity, with associations varying in direction and magnitude across categories. The association observed for Romani women was particularly large (*aOR* = 6.226), although this estimate should be interpreted with caution given the small size of this group (*n* = 8).

Regarding obstetric and healthcare characteristics, type of birth showed the largest associations, particularly for instrumental birth (*aOR* = 2.763). Private healthcare was associated with lower odds of perceived or suspected obstetric violence than public healthcare. Significant overall differences were also observed across Autonomous Communities (*p* = 0.031). Using Catalonia as the reference category, Galicia showed significantly higher odds of perceived or suspected obstetric violence, whereas Navarre showed significantly lower odds. No statistically significant differences were observed for the remaining Autonomous Communities compared with Catalonia. Full model results are presented in Table 3.

In the sensitivity analysis excluding participants who selected “do not know/no answer” (NK/NA), 5531 participants with complete data were included. The overall pattern of associations was consistent with the primary analysis. Full results are provided in Supplementary Table S2.

## 4. Discussion

In this sample of 6060 women, 33.5% reported or suspected obstetric violence. After mutual adjustment, perceived or suspected obstetric violence was associated with age, occupation, educational level, ethnicity, type of birth, type of healthcare and autonomous community, but not with social class. Type of birth showed the largest associations, whereas the explanatory capacity of the model was low (*Nagelkerke R*^2^ = 0.071). These associations are interpreted through an intersectional lens, although the additive model does not test intersectional effects.

Regarding the overall figure for obstetric violence, it is important to note that it was measured using a single, direct self-report question and that “yes” and “NK/NA” responses were combined, so it reflects perceived or suspected rather than confirmed obstetric violence. Because obstetric violence is a structural and highly normalized form of gender-based violence [19,25,28], many women may not identify it, and the figure may therefore underestimate its real extent in some groups. The results found as an overall result coincide with data previously found in the same context [17].

The results highlight the importance of maternal age. The percentage of women reporting or suspecting obstetric violence decreased progressively with age (43.1% at 18–25 years, 34.7% at 26–35, 31.2% at 36–45 and 23.8% above 45), in accordance with other studies [25,30,31]. In the adjusted model, participants aged 36–45 years had lower odds than those aged 26–35 years (*aOR* = 0.85), whereas the higher estimate among participants aged 18–25 (*aOR* = 1.49) years did not reach statistical significance (*p* = 0.056). These findings suggest age-related differences in perception, but the underlying mechanisms cannot be established because potentially relevant variables, such as parity, were not recorded. One possible explanation is that younger women may have less knowledge of childbirth-related rights and may be more exposed to paternalistic attitudes that infantilize women and limit their autonomy [15,32,33,34]; this hypothesis requires specific investigation.

Maternal occupation also reveals a significant association. Specifically, the present study shows that students (*aOR* = 2.16), housewives (*aOR* = 1.32) and self-employed women (*aOR* = 1.22) had higher odds of perceiving obstetric violence than employed women; the estimate for unemployed women was similar but not statistically significant (*aOR* = 1.21; *p* = 0.056). The pattern is heterogeneous and cannot be attributed to a single mechanism. It may reflect, in part, job instability or lower economic resources, which could expose women to less respectful care, although social class was not associated with the outcome. This could be framed within Bourdieu’s theory of social and cultural reproduction [35], according to which social inequalities are reproduced through institutions such as education and work, conditioning access to resources and opportunities. In the context of childbirth, women in less favourable circumstances may face additional barriers to accessing quality care that respects their rights, or may be treated differently by health professionals. These findings are partially in line with other studies [21,25,31,36,37], which report that obstetric violence is more frequent among women of lower socioeconomic status, and underline the need to address the structural inequities that perpetuate obstetric violence.

The ethnicity variable also plays an important role. In the adjusted model, the odds were higher among Romani women (*n* = 8; *aOR* = 6.23) and women of other ethnicities (*n* = 103; *aOR* = 1.51), whereas the estimate for Black women (*n* = 11; *aOR* = 1.91; *95*% *CI* 0.56–6.47) was in the same direction but not statistically significant. This is consistent with previous literature reporting higher rates of obstetric violence among ethnic minorities [38,39]. However, the sample sizes were very small and the estimates highly imprecise (e.g., Romani, *95*% *CI* 1.17–33.0), so these findings should be interpreted with great caution and their generalizability is limited. Despite this limitation, they underscore the importance of addressing cultural barriers and discrimination in medical care settings. Our study did not collect data on migrant status or country of birth, and ethnicity should not be equated with migration status (for instance, a large proportion of the Romani population in Spain holds Spanish nationality). Our data therefore do not allow conclusions about migrant women. Nevertheless, the literature on migrant women provides a useful conceptual framework to reflect on how racialized and ethnic inequalities may operate in maternity care. Wolf [40] argues that confronting obstetric violence against migrant women requires reconceptualising its nature, and proposes that it can be understood as a type of migratory injustice. From this perspective, obstetric violence is not confined to the health sphere but may also function as an instrument of oppression and discrimination, rooted in wider social structures of inequality and marginalization. The higher odds observed among women from ethnic minorities of obstetric violence may be interpreted from the perspective of intersectionality theory [16]. This theory posits that racism, sexism and ethnic discrimination interact and overlap, creating unique experiences of disadvantage for women belonging to multiple marginalized groups. In obstetric care, cultural, linguistic and ethnic barriers may lead to stereotyping, prejudice and a lack of cultural understanding among health professionals, resulting in inadequate treatment during childbirth. Given the minimal representation of these groups in our sample, Future research should use dedicated sampling strategies and record ethnicity and migrant status/country of birth as separate variables.

Type of birth is also associated with perception of obstetric violence. Compared with vaginal birth, the odds were higher for scheduled caesarean section (*aOR* = 1.40), urgent caesarean section (*aOR* = 2.16) and instrumental birth (*aOR* = 2.76), showing a gradient towards the most interventionist and unplanned modes of birth. This suggests an association between invasive medical procedures during delivery and women’s perception of obstetric violence, although the cross-sectional design does not allow causal conclusions. This perception reflects the need to review obstetric practices, especially those related to interventionism and medicalization, and to ensure that care is centred on the woman, recognizing her autonomy and rights during labour and birth [41]. However, type of birth alone does not indicate that a procedure was unnecessary, non-consensual or violent. The observed associations may reflect clinical complexity, urgency, unexpected changes in the birth plan, communication difficulties, reduced opportunities for shared decision-making or participants’ experiences of informed consent.

Analysis of the phenomenon of the medicalization of childbirth and the dominant technocratic model in obstetric care align with the ideas and concepts developed by the American anthropologist Robbie Davis-Floyd [42]. Her studies highlight how the introduction of medical technologies and interventionist procedures in childbirth care have transformed the experience of delivery and influenced women’s perceptions of the care they receive during the process of labour and birth. Davis-Floyd argues that the medicalization of childbirth has led to an increase in the use of medical interventions, such as inducing labour, administering analgesics, and performing caesarean sections and other instrumental procedures, which can contribute to women’s perception of obstetric violence. Her work emphasizes the importance of questioning the dominant obstetric practices and promoting a more respectful approach centred on the needs and wishes of women during childbirth [43]. Our findings are consistent with these contributions.

Type of healthcare was also associated with perception of obstetric violence: women attended in private healthcare reported it less often than those in public healthcare (27.7% vs. 33.8%), and had lower adjusted odds (*aOR* = 0.67), whereas receiving both types of care showed no difference. Lower perception does not necessarily imply lower occurrence, and it may reflect differences in expectations, continuity of care or user profile, which could not be explored in our data [27,44]. Since the level of the maternity unit was not recorded, differences between sectors should be interpreted with caution.

Geographic location was also associated with perceived or suspected obstetric violence. Galicia showed the highest percentage (41.3%). In the multivariable model, using Catalonia as the reference category, Galicia showed significantly higher adjusted odds of perceived or suspected obstetric violence (*aOR* = 1.285), whereas Navarre showed significantly lower adjusted odds (*aOR* = 0.546). No statistically significant differences were observed for the remaining autonomous communities compared with Catalonia. Since Catalonia was the reference category, differences between the other autonomous communities cannot be directly inferred from the reported comparisons, and several estimates are imprecise because of small samples. It should be remembered that Spain is divided into 17 autonomous communities and 2 autonomous cities (Ceuta and Melilla), each with exclusive responsibilities in health management; the autonomous cities are not represented in our data. This may suggest an influence of structural and contextual factors on the quality of perceived obstetric care, and the decentralization of health management could contribute to such differences [45]. The regional findings should be interpreted cautiously because recruitment was non-probabilistic, some regional samples were small and organizational characteristics of the healthcare services were not collected. The results do not establish that decentralization, regional health governance or specific clinical practices caused the observed differences.

The explanatory capacity of the model was low (*Nagelkerke R*^2^ = 0.071), indicating that the characteristics examined explain only a small part of the variability in perceived obstetric violence. This is consistent with obstetric violence being a structural and institutionalized phenomenon that cannot be explained solely by individual characteristics. The multivariable analysis should therefore be understood as providing evidence of adjusted associations, not as identifying a risk profile.

These findings are consistent with the proposal by Krieger [46], who argued that disparities in care and the perception of care may arise from the complex interaction between multiple factors that go beyond education or social class alone. She proposed a more holistic approach that considers social determinants of health in their entirety, including factors such as race, gender, ethnicity, geography, and political and socioeconomic history [46]. Similarly, in the context of perceived obstetric violence, social class showed no association in either the bivariate or the adjusted analysis, and educational level was associated with the outcome only after adjustment, with lower odds among women with primary education than among those with third-level education (*aOR* = 0.41; *n* = 60). This suggests that perception does not depend solely on material position and may also depend on the capacity to recognize and name mistreatment, which could be shaped by its normalization; this interpretation is tentative. Other factors, such as institutional culture, dominant obstetric practices, power relationships within the healthcare system and women’s individual experiences during childbirth, may also have a significant impact on how the quality and respectfulness of care are perceived.

Thus, the study findings support the need for a broader, multidimensional understanding of obstetric violence. The low explanatory capacity of the model is consistent with under-standing obstetric violence as a structural and gender-related phenomenon that cannot be explained solely by individual sociodemographic or obstetric characteristics. In this framework, the factors identified in the multivariable analysis should be interpreted as characteristics associated with differences in the perception of obstetric violence rather than as diagnostic or predictive markers. Since all participants in the present study were women, the effect of gender itself could not be directly estimated in the model and should therefore be understood as part of the conceptual framework rather than as an association demonstrated by the present analysis. Further studies incorporating individual, organizational, healthcare, and broader structural determinants are needed to better understand the mechanisms underlying obstetric violence.

Although our sample is not representative of all women in Spain, our results are consistent with the 2024 State of World Population report by the United Nations Population Fund, which warns that the international community has focused on the groups that are easiest to reach and has neglected inequalities in systems and societies, so that slight gradients become steep drops [47].

Finally, it should be noted that this cross-sectional study sheds light on the complexity of the factors that influence the perception of obstetric violence. However, to fully comprehend these associations and address the disparities identified in obstetric health care, further in-depth research is required, perhaps using a qualitative approach. Such an approach could offer a richer, contextualized understanding of women’s experiences during childbirth and of the structural and sociocultural factors that contribute to the perception of obstetric violence in different population groups, and could help to inform interventions that respond to the needs of different groups of women, thus contributing to more equitable, respectful obstetric care for all women.

### 4.1. Limitations

This study presents several limitations that must be acknowledged. First, the sample selection was non-probabilistic and conducted primarily through social networks and birth support groups, such as the Obstetric Violence Observatory and the Birth is Ours association. This convenience sampling strategy introduces a significant selection bias, as women affiliated with or following these networks may have a higher baseline awareness of, or sensitization to, obstetric violence compared to the general population. Participation was also possible only through an online questionnaire that required signing in with an e-mail account, which excludes women without internet access, digital skills or an e-mail account, and may contribute to the under-representation of some groups. In addition, some births occurred up to about 13 months before the survey, so responses may be affected by recall bias, and the time elapsed since birth was not recorded. The data were collected during the COVID-19 pandemic (see Methods), although previous studies by our group yielded very similar results before and during the pandemic [17,18]. Duplicate control relied on a single response per e-mail account and cannot completely exclude multiple submissions by the same woman using different accounts; women with two births within the eligibility window could report only one of them, so the second birth was not captured.

Second, obstetric violence was measured with a single, direct self-report item, which captures women’s perception rather than specific practices, does not distinguish between types or severity of mistreatment, and depends on how each woman understands the term and on the degree to which mistreatment has been normalized. It may therefore underestimate the true extent of the phenomenon in some groups.

Third, as this is a secondary analysis of an existing dataset, the variables available were those collected in the original survey. Relevant variables such as parity, time since birth, level of the maternity unit and migrant status/country of birth were therefore not available, so residual confounding cannot be ruled out; future studies should record them, together with type and degree of disability. The explanatory capacity of the model was low (*Nagelkerke R*^2^ = 0.071).

Fourth, despite the study’s focus on intersectionality and its large sample size, there is a substantial lack of representativeness regarding sociodemographic diversity. The sample features a strong overrepresentation of women who identify as white or Caucasian (98%, *n* = 5887), with third-level education (78.2%, *n* = 4736), and from the middle class (93.5%, *n* = 5664). In particular, the very small sample sizes of ethnic minorities, such as Romani (*n* = 8) and Black women (*n* = 11), mean that the regression results for ethnicity must be interpreted with great caution. The estimate for Romani women (a*OR* = 6.23; *95*% *CI* 1.17–33.0) is highly imprecise and may be unstable owing to sparse data, so it should not be considered a reliable estimate of the magnitude of the association. Likewise, other groups were small (e.g., primary education, *n* = 60; students, *n* = 55; La Rioja, *n* = 37), and multi-categorical analyses lack adequate statistical power for underrepresented intersections, which is why interaction terms were not estimated. Consequently, our findings primarily describe a non-generalizable profile centred on women who are mostly white, highly educated and middle class. Future research should consider probabilistic sampling and targeted recruitment of these groups through two complementary pathways: (a) intra-group studies dedicated to specific populations (e.g., Romani-only or Black-only cohorts) that explore heterogeneity, structural discrimination and institutional mistrust without treating minority experiences as deviations from a white normative group [48,49], and (b) stratified probabilistic designs with oversampling of racialized and ethnic minority women, which may allow adequately powered intersectional models [50,51]. Qualitative and mixed studies, in line with Crenshaw’s view of intersectionality as fluid and interactive, would also help to confirm and deepen our findings.

### 4.2. Implications for Health Policy and Clinical Practice

First, perceived or suspected obstetric violence was substantially more frequent after urgent caesarean section and instrumental birth than after vaginal birth. These are situations in which time pressure may reduce opportunities for explanation and consent, so units could consider and evaluate strategies that reinforce continuous information, real-time verbal explanations and informed consent during unplanned or instrumental births [52,53,54]. Second, a considerable proportion of the women reported or suspected obstetric violence, and “NK/NA” responses were included in the outcome because they may, in part, reflect unrecognized mistreatment. Together with the lower odds observed among women with primary education, this is compatible with a normalization of mistreatment that hinders its recognition. Routine measurement of women’s experience of care with standardized instruments, and antenatal education on childbirth rights and respectful care, could help to make this phenomenon visible. Third, we observed differences between autonomous communities, with Galicia showing the highest percentage and most other communities showing lower adjusted odds than Galicia. As our data measure perception and cannot identify the reasons for these differences, we suggest using homogeneous indicators of women’s experience across territories to monitor and understand them before establishing common standards of practice.

## 5. Conclusions

In conclusion, this study shows that perceived or suspected obstetric violence is associated with several sociodemographic, obstetric and healthcare characteristics, which supports considering multiple social positions and clinical characteristics within an intersectionally informed framework. These associations were mutually adjusted, but the low explanatory capacity of the model indicates that the characteristics examined explain only a small part of the phenomenon, consistent with its structural nature. Type of birth showed the strongest associations: perceived or suspected obstetric violence was more frequent after urgent caesarean section and, especially, after instrumental birth than after vaginal birth. The association between type of birth and the perception of obstetric violence emphasizes the need to review obstetric practices, focusing on respect for women’s autonomy. Age, occupation, ethnicity, type of healthcare and autonomous community were also associated with the outcome, although these associations were heterogeneous across categories and, in the case of ethnicity, based on very small groups. Educational level was associated with perception only after adjustment, with lower odds among women with primary education, whereas social class was not associated with the outcome. This underscores the complexity of the factors that influence the perception of obstetric violence and is compatible with difficulties in recognizing mistreatment that has become normalized.

## Figures and Tables

**Table 1:** Descriptive results of sociodemographic, obstetric, and obstetric violence variables (n = 6060).

Variable		% (n) ^1^	Variable		% (n) ^1^
Age (n = 6029)	18–25	1.9 (116)	Autonomous Community providing care	Andalusia	13.0 (789)
	26–35	58.1 (3502)		Aragón	4.7 (287)
	36–45	39.6 (2390)		Canary Islands	3.3 (202)
	>45	0.3 (21)		Cantabria	1.0 (59)
Occupation	Housewife	4.6 (279)		Castilla y León	5.9 (356)
	Student	0.9 (55)		Castilla-La Mancha	3.5 (212)
	Unemployed	9.6 (579)		Catalonia	14.1 (855)
	Employed	73.0 (4423)		Valencian C.	14.7 (892)
	Self-employed	9.7 (586)		Extremadura	2.4 (146)
	Other	2.3 (138)		Galicia	8.2 (496)
Education level	Primary	1.0 (60)		Murcia Region	2.7 (161)
	Secondary	20.9 (1264)		La Rioja	0.6 (37)
	Third level	78.2 (4736)		Madrid	15.7 (949)
Social class	Low	4.3 (260)		Navarre	1.4 (85)
	Middle	93.5 (5664)		Basque Country	4.8 (292)
	High	2.2 (136)		Principality of Asturias	1.8 (107)
Ethnicity (n = 6009)	White or Caucasian	98.0 (5887)		Balearic Islands	2.2 (135)
	Romani	0.1 (8)			
	Black	0.2 (11)	Type of healthcare	Private	9.6 (584)
	Other	1.7 (103)		Public	58.9 (3572)
Type of birth	Sched C-sec	6.5 (395)		Both	31.4 (1904)
	Urgent C-sec	15.1 (914)	Obstetric Violence	No	66.5 (4030)
	Instrumental	14.3 (869)		Yes or suspected	33.5 (2030)
	Vaginal	64.1 (3882)			

^1^: *n* = frequencies.

**Table 2:** Bivariate results of sociodemographic and obstetric variables related to obstetric violence.

Variables		Perceived Obstetric Violence		
		No	Yes or Suspected	
		% (n) ^1^	% (n) ^1^	*p*-Value ^2^
Age	18–25	56.9 (66)	43.1 (50)	0.003
	26–35	65.3 (2286)	34.7 (1216)	
	36–45	68.8 (1645)	31.2 (745)	
	>45	76.2 (16)	23.8 (5)	
Occupation	Housewife	64.2 (179)	35.8 (100)	0.003
	Student	50.9 (28)	49.1 (27)	
	Unemployed	62.9 (364)	37.1 (215)	
	Employed	67.7 (2996)	32.3 (1427)	
	Self-employed	62.6 (367)	37.4 (219)	
	Other	69.6 (96)	30.4 (42)	
Education level	Primary	78.3 (47)	21.7 (13)	0.129
	Secondary	67 (847)	33 (417)	
	Third level	66.2 (3136)	33.8 (1600)	
Social class	Low	61.2 (159)	38.8 (101)	0.175
	Middle	66.7 (3780)	33.3 (1884)	
	High	66.9 (91)	33.1 (45)	
Ethnicity	White or Caucasian	66.7 (3926)	33.3 (1961)	0.004 ^3^
	Romani	25 (2)	75 (6)	
	Black	45.5 (5)	54.5 (6)	
	Other	56.3 (58)	43.7 (45)	
Type of birth	Scheduled C-section	67.6 (267)	32.4 (128)	<0.001
	Urgent C-section	55.8 (510)	44.2 (404)	
	Instrumental	50.1 (435)	49.9 (434)	
	Vaginal	72.6 (2818)	27.4 (1064)	
Type of healthcare	Private	72.3 (422)	27.7 (162)	0.007
	Public	66.2 (2363)	33.8 (1209)	
	Both	65.4 (1245)	34.6 (659)	

^1^: *n* = frequencies; ^2^: Chi-square test; ^3^: Fisher’s exact test.

**Table 3:** Multivariable binary logistic regression of factors associated with perceived or suspected obstetric violence.

Predictor	Coef. ^1^	SE ^2^	aOR ^3^	CI95% ^4^	*P* ^5^	Adjusted GVIF ^6^
Constant	−0.640	0.106	0.527	0.428–0.649	<0.001	
Age					0.003	
26–35 ^†^			1.000			1.018
18–25	+0.398	0.209	1.489	0.989–2.240	0.056	
36–45	−0.168	0.059	0.846	0.753–0.949	0.004	
>45	−0.550	0.524	0.577	0.207–1.612	0.294	
Ethnicity					0.023	
White/Caucasian ^†^			1.000			1.006
Romani	1.829	0.851	6.226	1.174–33.03	0.032	
Black	0.645	0.624	1.906	0.562–6.471	0.301	
Other	0.415	0.208	1.514	1.006–2.277	0.046	
Social class					0.265	
Middle ^†^			1.000			1.018
High	−0.054	0.192	0.947	0.650–1.380	0.779	
Low	0.222	0.139	1.249	0.951–1.640	0.109	
Type of birth					<0.001	
Vaginal ^†^			1.000			1.010
Scheduled C-section	0.335	0.117	1.398	1.112–1.758	0.004	
Urgent C-section	0.770	0.078	2.159	1.854–2.515	<0.001	
Instrumental	1.016	0.079	2.763	2.368–3.224	<0.001	
Occupation					0.006	
Employed ^†^			1.000			1.016
Housewife	0.275	0.137	1.316	1.006–1.722	0.045	
Student	0.769	0.285	2.157	1.235–3.769	0.007	
Other	−0.056	0.196	0.945	0.644–1.387	0.773	
Unemployed	0.188	0.098	1.207	0.995–1.464	0.056	
Self-employed	0.198	0.095	1.219	1.012–1.469	0.037	
Type of healthcare					<0.001	
Public ^†^			1.000			1.029
Private	−0.406	0.106	0.666	0.541–0.820	<0.001	
Both	0.009	0.063	1.009	0.891–1.143	0.883	
Education level					0.009	
Third level ^†^			1.000			1.033
Primary	−0.884	0.342	0.413	0.211–0.808	0.010	
Secondary	−0.138	0.073	0.872	0.755–1.007	0.061	
Autonomous Community					0.031	
Catalonia ^†^			1.000			
Andalusia	−0.154	0.109	0.859	0.692–1.062	0.159	1.005
Aragón	0.105	0.149	1.111	0.829–1.489	0.481	
Canary I.	−0.285	0.179	0.752	0.530–1.067	0.110	
Cantabria	−0.205	0.299	0.815	0.454–1.463	0.493	
Castilla y León	−0.111	0.140	0.895	0.680–1.178	0.427	
Castilla-La Mancha	0.139	0.165	1.149	0.832–1.587	0.399	
Valencian C.	−0.182	0.107	0.834	0.676–1.028	0.088	
Extremadura	0.131	0.193	1.140	0.781–1.666	0.497	
Galicia	0.251	0.122	1.285	1.011–1.633	0.040	
Balearic I.	−0.061	0.206	0.941	0.629–1.407	0.766	
La Rioja	0.207	0.359	1.230	0.608–2.487	0.564	
Madrid	−0.010	0.104	0.990	0.808–1.213	0.924	
Navarre	−0.606	0.283	0.546	0.313–0.951	0.033	
Basque C.	−0.115	0.152	0.892	0.662–1.201	0.450	
P. Asturias	−0.214	0.229	0.808	0.515–1.265	0.351	
Murcia R.	−0.044	0.188	0.957	0.662–1.383	0.815	

^1^: Coefficient; ^2^: Standard error; ^3^: Adjusted Odds Ratio, for the constant, Exp(B) represents the baseline odds; ^4^: Confidence interval 95%; ^5^: *p*-value; ^6^: Collinearity statistics; ^†^: Reference category.

## Data Availability

The datasets used and/or analysed during the current study are available from the corresponding author on reasonable request.

## Supplementary Materials

The following supporting information can be downloaded at: https://www.mdpi.com/article/10.3390/nursrep16100366/s1, Table S1: Sensitivity analysis comparing multivariable logistic regression models according to the classification of perceived obstetric violence; Table S2: Original response categories of perceived obstetric violence (no, do not know, yes).

## Public Involvement Statement

No public involvement in any aspect of this research.

## Guidelines and Standards Statement

This manuscript was drafted against the STROBE checklist for cross-sectional research.
