# Supplementary material for: Sociodemographic and Perinatal Correlates of Perceived Obstetric Violence in Spain: A Cross-Sectional Study Informed by an Intersectional Framework
Source: Nurs Rep. 2026 Oct 15;16(10):366. doi: 10.3390/nursrep16100366 (PMC13708642; doi:10.3390/nursrep16100366)
Supplement: Supplementary file 1 [file nursrep-16-00366-s001.zip › nursrep-4587621-supplementary.pdf]

Table S1. Sensitivity analysis comparing multivariable logistic regression models according to the classification of perceived obstetric violence.

	Primary analysis		Sensitivity analysis	
**Predictor**	**aOR** [1]	**CI95%**[2]	**aOR**[1]	**CI95%**[2]
**Age**				
26-35†				
18-25	1.489	0.989-2.240	0.806	
36-45	0.846	0.753-0.949	0.678	
>45	0.577	0.207-1.612	0.233	
**Ethnicity**				
White/Caucasian†				
Romani	6.226	1.174-33.03	7.388	
Black	1.906	0.562-6.471	2.142	
Other	1.514	1.006-2.277	1.593	
**Social class**				
Middle†				
High	0.947	0.650-1.380	1.046	
Low	1.249	0.951-1.640	1.125	
**Type of birth**				
Vaginal†				
Scheduled C-section	1.398	1.112-1.758	1.496	
Urgent C-section	2.159	1.854-2.515	2.263	
Instrumental	2.763	2.368-3.224	3.073	
**Occupation**				
Employed†				
Housewife	1.316	1.006-1.722	1.380	
Student	2.157	1.235-3.769	2.090	
Other	0.945	0.644-1.387	0.808	
Unemployed	1.207	0.995-1.464	1.300	
Self-employed	1.219	1.012-1.469	1.247	
**Type of healthcare**				
Public†				
Private	0.666	0.541-0.820	0.653	
Both	1.009	0.891-1.143	0.971	
**Education level**				
Third level†				
Primary	0.413	0.211-0.808	0.378	
Secondary	0.872	0.755-1.007	0.851	
**Autonomous Community**				
Cataluña†				
Andalusia	0.859	0.692-1.062	0.816	0.645-1.031
Aragón	1.111	0.829-1.489	1.061	0.770-1.461
Canary I.	0.752	0.530-1.067	0.812	0.558-1.180
Cantabria	0.815	0.454-1.463	0.897	0.486-1.657
Castilla y León	0.895	0.680-1.178	0.837	0.618-1.134
Castilla-La Mancha	1.149	0.832-1.587	1.190	0.843-1.680
Valencian C.	0.834	0.676-1.028	0.782	0.622-0.984
Extremadura	1.140	0.781-1.666	1.033	0.679-1.572
Galicia	1.285	1.011-1.407	1.335	1.033-1.726

Balearic I.	0.941	0.629-1.407	0.988	0.641-1.522
La Rioja	1.230	0.608-2.487	1.376	0.656-2.889
Madrid	0.990	0.808-1.213	0.899	0.719-1.124
Navarre	0.546	0.313-0.951	0.528	0.286-0.977
Basque C.	0.892	0.662-1.201	0.886	0.641-1.224
P. Asturias	0.808	0.515-1.265	0.814	0.502-1.321
Murcia R.	0.957	0.662-1.383	0.894	0.595-1.343

1: Adjusted Odds Ratio, for the constant, Exp(B) represents the baseline odds; 2: Confidence interval 95%

Table S2. Original response categories of perceived obstetric violence (no, do not know, yes).

Variables		Perceived Obstetric Violence			
		No	NK/NA	Yes	
		%(n)[1]	%(n)[1]	%(n)[1]	*p*-value[2]
**Age**	18-25	56.9(66)	13.8(16)	29.3(34)	0.002[3]
	26-35	65.3(2286)	7.4(260)	27.3(956)	
	36-45	68.8(1645)	7.2(171)	24(574)	
	>45	76.2(16)	14.3(3)	9.5(2)	
**Occupation**	Housewife	64.2(179)	7.5(21)	28.3(79)	0.020
	Student	50.9(28)	12.7(7)	36.4(20)	
	Unemployed	62.9(364)	7.3(42)	29.9(173)	
	Employed	67.7(2996)	7.3(323)	25(1104)	
	Self-employed	62.6(367)	7.8(46)	29.5(173)	
	Other	69.6(96)	9.4(13)	21(29)	
**Education level**	Primary	78.3(47)	8.3(5)	13.3(8)	0.231
	Secondary	67(847)	7.6(96)	25.4(321)	
	Third level	66.2(3136)	7.4(351)	26.4(1249)	
**Social class**	Low	61.2(159)	11.5(30)	27.3(71)	0.076
	Middle	66.7(3780)	7.3(415)	25.9(1469)	
	High	66.9(91)	5.1(7)	27.9(38)	
**Ethnicity**	White or Caucasian	66.7(3926)	7.4(438)	25.9(1523)	0.032[3]
	Romani	25(2)	12.5(1)	62.5(5)	
	Black	45.5(5)	9.1(1)	45.5(5)	
	Other	56.3(58)	8.7(9)	35(36)	
**Type of birth**	Scheduled C-section	67.6(267)	7.1(28)	25.3(100)	<0.001
	Urgent C-section	55.8(510)	9.4(86)	34.8(318)	
	Instrumental	50.1(435)	8.5(74)	41.4(360)	
	Vaginal	72.6(2818)	6.8(264)	20.6(800)	
**Type of healthcare**	Private	72.3(422)	6.5(38)	21.2(124)	0.019
	Public	66.2(2363)	7.2(257)	26.7(952)	
	Both	65.4(1245)	8.2(157)	26.4(502)	

1: n=frequencies; 2: Chi-square test; 3: Fisher's exact test

**Obstetric Violence**	No	66.5(4030)
	Don't know	7.5(452)
	Yes	26.0(1578)
